# Growth impairment in children with atrophic autoimmune thyroiditis and pituitary hyperplasia

**DOI:** 10.1186/s13052-024-01641-w

**Published:** 2024-04-23

**Authors:** Domenico Corica, Tiziana Abbate, Anna Malgorzata Kucharska, Malgorzata Wojcik, Francesco Vierucci, Mariella Valenzise, Alessandra Li Pomi, Giorgia Pepe, Gerdi Tuli, Beata Pyrzak, Tommaso Aversa, Malgorzata Wasniewska

**Affiliations:** 1 Department of Human Pathology of Adulthood and Childhood, Unit of Pediatrics, University of Messina, Via Consolare Valeria 1, 98125 Messina, Italy; 2 Department of Pediatrics and Endocrinology, Medical University of Warsaw, Warsaw, Poland; 3 Department of Pediatric and Adolescent Endocrinology, Chair of Pediatrics, Pediatric Institute, Jagiellonian University Medical College, Kraków, Poland; 4 Pediatric Unit, San Luca Hospital, Lucca, Italy; 5 Department of Public Health and Paediatric Sciences, Paediatric Endocrinology, University of Turin, Regina Margherita Children’s Hospital, Turin, Italy

**Keywords:** Adenohypophysis hyperplasia, Autoimmune thyroiditis, Hypothyroidism, Growth arrest, Children

## Abstract

**Background:**

Atrophic autoimmune thyroiditis (AAT) is a rare phenotype of autoimmune thyroiditis (AT) in pediatric age. AAT occurs without thyroid enlargement leading to a delay in its diagnosis. Growth impairment is infrequent in autoimmune thyroiditis, if timely diagnosed. Prolonged severe hypothyroidism is a rare cause of pituitary hyperplasia (PH) in childhood. Loss of thyroxine negative feedback causes a TRH-dependent hyperplasia of pituitary thyrotroph cells resulting in adenohypophysis enlargement. A transdifferentiation of pituitary somatotroph cells into thyrotroph cells could explain growth failure in those patients.

**Methods:**

Twelve patients were retrospectively evaluated at five Italian and Polish Centres of Pediatric Endocrinology for height growth impairment. In all Centres, patients underwent routine clinical, biochemical and radiological evaluations.

**Results:**

At the time of first assessment, the 75% of patients presented height growth arrest, while the remaining ones showed growth impairment. The study of thyroid function documented a condition of hypothyroidism, due to AT, in the entire cohort, although all patients had no thyroid enlargement. Thyroid ultrasound showed frankly atrophic or normal gland without goiter. Cerebral MRI documented symmetrical enlargement of the adenohypophysis in all patients and a homogeneous enhancement of the gland after the administration of Gadolinium-DPTA. Replacement therapy with levothyroxine was started and patients underwent close follow-up every 3 months. During the 12 months of follow-up, an improvement in terms of height growth has been observed in 88% of patients who continued the follow-up. Laboratory findings showed normalization of thyroid function and the control brain MRI documented complete regression of PH to a volume within the normal range for age and sex.

**Conclusions:**

This is the largest pediatric cohort with severe autoimmune primary hypothyroidism without goiter, but with pituitary hyperplasia in which significant growth impairment was the most evident presenting sign. AAT phenotype might be correlated with this specific clinical presentation. In youths with growth impairment, hypothyroidism should always be excluded even in the absence of clear clinical signs of dysthyroidism.

**Supplementary Information:**

The online version contains supplementary material available at 10.1186/s13052-024-01641-w.

## Introduction

Autoimmune thyroiditis (AT) is the most common cause of primary hypothyroidism (PHT) in children and adolescents. Atrophic autoimmune thyroiditis (AAT) is a particular form of AT whose incidence is rare in children [1]. Unlike the classic phenotype, AAT occurs with hypothyroidism without enlargement of the thyroid gland. The absence of goiter may delay the diagnosis, causing severe hypothyroidism and growth impairment [2–4].

Long-standing primary hypothyroidism is an unusual cause of pituitary hyperplasia (PH) in children. In PHT, loss of thyroxine feedback determines an overproduction of thyrotropin releasing hormone (TRH), followed by hyperplasia of thyrotropin (TSH)-releasing cells in the anterior pituitary and by a consequent pituitary enlargement [5–6]. There are various causes of sellar mass in children such as pituitary adenomas, suprasellar pituitary tumors, craniopharyngeal neoplasm and intracranial germ cell tumors that can occur with overlapped clinical pictures. It might be difficult to distinguish PH from pituitary adenomas on brain computed tomography (CT) or magnetic resonance imaging (MRI), contrary to the other causes that can be safely excluded [7]. Adequate treatment of PHT with Levothyroxine replacement therapy has been shown to determine regression of the PH in about 76% of cases [7, 8].

In this international multicentre study, we report on a case series of patients diagnosed with pituitary hyperplasia due to severe acquired autoimmune hypothyroidism without goiter, with a presenting picture characterized by height growth impairment.

## Materials and methods

All patients diagnosed with PH due to autoimmune PHT were retrospectively evaluated at the five Italian and Polish Centres of Pediatric Endocrinology.

In all participating Centres, patients underwent routine clinical, biochemical and radiological evaluations. Clinical evaluation was performed in accordance with standardized procedures [9]. Height was measured to the nearest 0.1 cm on standardized, wall-mounted height boards, according to standardized procedures. The children stood with the head aligned in the Frankfort plane, barefoot, with feet placed together and flat on the ground, heels, buttocks, and scapulae against the vertical backboard, arms loose and relaxed with the palms facing medially. Body weight was determined to the nearest 0.1 kg on accurate and properly calibrated standard beam scales, in minimal underclothes and no shoes. BMI was calculated using the equation: body weight (kg)/height (m)2. The subjects underwent a detailed physical examination and pubertal evaluation, assessed by five Tanner stages of breast development in girls and testicular volume in boys. Bone age was assessed by expert pediatric endocrinologists’ evaluation of a left wrist x-ray compared with the standardized bone age from the Greulich and Pyle atlas.

Height growth arrest (stunting) was defined as height growth < 1 cm over 6 months in the presence of epiphyses not welded at the bone age evaluation; poor height growth was defined as height growth velocity/year ≤ -1.0 SDS for age and sex assessed at least 6 months apart or a reduction in stature of 0.5 SDS/year for age in children older than two years.

Biochemical investigations consisted of assessing thyroid function by measuring the levels of thyroid hormones (free triiodothyronine or FT3 and free-thyroxine or FT4), thyroid stimulating hormone (TSH), thyroid peroxidase antibodies (TPOAb) and thyroglobulin antibodies (TGAb). In addition, hormone secretion of the adenohypophysis was assessed by assay of adrenocorticotropic hormone (ACTH), prolactin (PRL), follicle stimulating hormone (FSH) and luteinizing hormone (LH). Levels of other hormones were also assessed including estradiol, testosterone, morning cortisol and insulin-like growth factor-1 (IGF-1). The measurement of FT3, FT4, TSH, anti-thyroid antibodies, ACTH, cortisol, PRL, FSH, LH, testosterone, estradiol, IGF-1 was performed by chemiluminescence immunoassay methods at each center. Hormone and anti-thyroid antibody values were converted to the same reference unit of measurement if necessary.

Some patients in whom growth hormone (GH) deficiency was suspected after restoring normal thyroid function, were tested for GH secretion by a standard stimulation test with clonidine and glucagon at the same Italian centre. The clonidine stimulation test consisted of oral administration of clonidine at a dose of 0.15 mg/m2 (max 150 mcg) and the later measurement of the levels of growth hormone every 30 min for a total of 2 h. The glucagon stimulation test consisted of intramuscular administration of glucagon at a dose of 0.1 mg/Kg (max 1 mg) and the later measurement of the levels of growth hormone every 30 min for a total of 3 h [10]. After stimulation, a peak of GH ≥ 8 ng/ml is considered normal [11].

Moreover, a blood sampling for plasma triglycerides, HDL cholesterol, LDL cholesterol, total cholesterol, glutamate-oxaloacetate transaminase (GOT), glutamate-pyruvate transaminase (GPT) and creatine kinase was carried out.

Ultrasonography of the thyroid gland was performed with a particular attention to the thyroid volume and echogenicity. Thyroid color-doppler ultrasound was performed to evaluate thyroid vascularization.

All patients underwent cerebral MRI, also with the administration of Gadolinium-DTPA. Particular attention was focused on the study of the pituitary gland, whose volume was estimated by using the formula: V = Antero-posterior dimension X Craniocaudal dimension X Transverse dimension X 0.52 (this factor is obtained from the sphere volume equation coefficient and cubic volume calculation: (4/3π) (r3)/(2r)3 = 3.1416/6 = 0.52) [12].

## Results

Twelve Caucasian patients (6 females– 6 males) have been retrospectively evaluated in 5 Pediatric Endocrinology Centers from Italy and Poland. The reason for referral to the pediatric endocrinology outpatient clinic for all these patients were height growth arrest or growth impairment. In all patients, the clinical picture was characterized by the absence of goiter in the context of AAT.

The clinical characteristics of patients are reported in Table 1. At the time of the first assessment, the median age of the patients was 11.3 years (IQR 8–14.8), specifically 9.51 years in the female sex and 12.68 years in the male sex. Seven patients (58,3%) were prepubertal (Tanner stage 1). The median values of height and BMI were − 1.91 SDS (-3.58– +0.78) and + 1.12 SDS (0.01–3.1), respectively. Difference between the median values of height and target height was − 1.3 SDS. Bone age was delayed with respect to chronological age in almost the entire cohort (75%) with a median bone age retardation of 2.4 years (0-6.2). In particular, in patient 2, 9, 10 and 11, bone age was overlapped to the chronological age. Furthermore, it is important to note that patient 12 presented an extremely delayed bone age that is a typical feature of Van Wyk-Grumbach syndrome [6, 13, 14].

Hypokinesia (83%) and dry and thickened skin (83%) were the most reported clinical findings. Nine patients (75%) presented growth arrest, while the remaining cohort showed growth impairment.

**Table 1:** Clinical features at the diagnosis of primary hypothyroidism

Patients	Sex	Age (years)	Height, cm (SDS)	Target Height SDS	BMI, Kg/m2 (SDS)	Bone Age	Pubertal Tanner’s Stage (B/G-P)	Main symptoms and clinical findings
Patient 1	F	13	132.8 (-3.58)	-0.59	22.4 (1.08)	10.25	B2P1	GA, dry and thickened skin
Patient 2	F	11	130 (-2.3)	NA	20.41 (1.16)	11.3	B1P2	GA, hypokinetic, apathy, constipation
Patient 3	F	8	123.9 (-0.84)	-0.81	18.11 (1.06)	6.2	B1P1	GA, dry and thickened skin
Patient 4	M	11.7	135.2 (-1.67)	-0.9	23.5 (2.08)	8.6	G1P1	GA, hypokinetic, apathy, fatigue, constipation, sleepiness, impaired school performance, cold intolerance, bradycardia, sparse brittle hair, dry and thickened skin
Patient 5	M	14.8	151.3 (-2.09)	0.7	21.57 (0.71)	12.6	G2P2	GA, hypokinetic, apathy, fatigue, constipation, sleepiness, cold intolerance, bradycardia, sparse brittle hair, dry and thickened skin
Patient 6	F	8.5	113.3 (-2.7)	-0.6	17.7 (0.87)	3.6	B1P1	GA, hypokinetic, apathy, fatigue, constipation, sleepiness, cold intolerance, bradycardia, dry and thickened skin
Patient 7	F	8.5	119 (-1.74)	-0.6	15.9 (0.01)	5.6	B1P1	GA, hypokinetic, apathy, fatigue, constipation, sleepiness, cold intolerance, bradycardia, dry and thickened skin
Patient 8	M	11.1	129 (-2.19)	NA	21.6 (1.74)	6	G1P1	PG, hypokinetic, apathy, impaired school performance, bradycardia, sparse brittle hair, dry and thickened skin
Patient 9	F	8.1	123 (-0.75)	NA	21.5 (2.22)	8.1	B2P1	PG, hypokinetic, fatigue, cold intolerance, dry and thickened skin
Patient 10	M	11.5	151.5 (0.78)	0.4	29.8 (3.13)	11.5	G1P2	PG, hypokinetic, apathy, fatigue, impaired school performance
Patient 11	M	14.62	158.8 (-1.05)	NA	20 (0.22)	14	G5P4	GA, dry and thickened skin, hypokinetic, fatigue, sleepiness, cold intolerance
Patient 12	M	12.39	133 (-2.59)	NA	26.6 (2.46)	6.5	G4P2	GA, bradycardia, sparse brittle hair, dry and thickened skin, hypokinetic, fatigue, constipation, sleepiness, impaired school performance, cold intolerance

SDS = Standard Deviation score; GA = growth arrest; PG = poor linear growth; B = breast; G = gonads; P = pubic hair; M = male; F = female; NA = not available

Laboratory findings are summarized in Table 2. Hypothyroidism due to AT was demonstrated in the entire population. The median values of thyroid hormones and anti-thyroid antibodies were: TSH 981 mIU/L (IQR 236.6–1648), FT4 3.55 pmol/L (IQR 0.5–6.2), TPOAb 490.3 IU/ml (IQR 88.8–4580), TGAb 391 IU/ml (IQR 15.5–2040). Thyroid ultrasound showed frankly atrophic thyroid (41.6%) or thyroid gland size within normal limits without goiter (58.4%) associated with diffusely hypoechogenic, coarse and heterogeneous parenchymal echotexture. On thyroid color doppler ultrasound evaluation, there were no significant alterations. Evaluation of anterior pituitary hormone function showed increased PRL levels in 58,3% of patients with median value of 660 u[IU]/ml (IQR 214–1362). ACTH levels were within normal limits in all patients. FSH and LH were in prepubertal-range. The median value of gonadotropins was: FSH 3 mIU/ml (IQR 1.2–7.5) and LH 0.3 mUI/ml (IQR 0.1-3).

Low concentrations of cortisol were documented, instead, in 41,6% of patients with median value of 9 ug/dl (IQR 5.3–216.3). The values of insulin growth factor-1 (IGF-1) were lower than − 2 SDS in 42% of patients (median value 91 ng/ml; IQR: 53.6–201); the reference values for IGF-1 by sex and age range are given in supplementary material (see Table S1, Additional File1).

High levels of total cholesterol and LDL cholesterol were documented in 70% of patients, with a median value of 241.5 mg/dl and 170.4 mg/dl, respectively. In 50% of patients high levels of triglycerides (median 110 mg/dl) and of GOT and GPT (median value of 92 U/L and 63 U/L respectively) were observed.

**Table 2:** Laboratory findings and pituitary volume at the diagnosis of PH and AAT

Patients	TSH, mUI/ml (nv 0.27–4.2)	FT4, pmol/l (nv 12–22)	TPOAb, IU/ml (nv 0–34)	TGAb, IU/ml (nv 0-115)	IGF-1, ng/ml	PRL, uUI/ml (nv 86–496)	E2, pg/ml (pv < 12)	TEST, ng/dl (pv < 20)	Pituitary Volume, mm3
Patient 1	236.6	3.2	3486	200	53.6	214	13	NA	650
Patient 2	319	2.79	4580	289	88.5	497	5	NA	648,9
Patient 3	1214	1.03	597	1805	73.1	999	12.4	NA	514,8
Patient 4	962	5.5	1185.8	554.8	112	659.5	NA	NA	1830
Patient 5	1000	5.5	88.8	15.5	120.3	723.4	NA	NA	1965
Patient 6	1000	1.93	187.6	36.8	123.3	1362	NA	NA	1419,6
Patient 7	359.5	6.17	383.6	416.9	93.6	434	NA	NA	532,48
Patient 8	1648	3	1020	2040	NA	NA	NA	NA	1123
Patient 9	1113	4.3	1020	2040	55.8	838.2	20	NA	1514
Patient 10	324	0.5	98	31	201	255	NA	25	504
Patient 11	750	3.9	168	391	NA	485.8	16.4	418	3528
Patient 12	1016	4	296.4	NA	76.8	835.8	NA	NA	770

AAT = Atrophic autoimmune thyroiditis; PH = pituitary hyperplasia; TSH = thyroid stimulating hormone; FT4 = free thyroxine; TPOAb = thyroid peroxidase antibodies; TGAb = thyroglobulin antibodies; IGF-1 = insulin growth factor-1; PRL = prolactin; E2 = estradiol; TEST = testosterone; nv = normal values; NA = not available, pv = prepubertal values

**Fig. 1:**
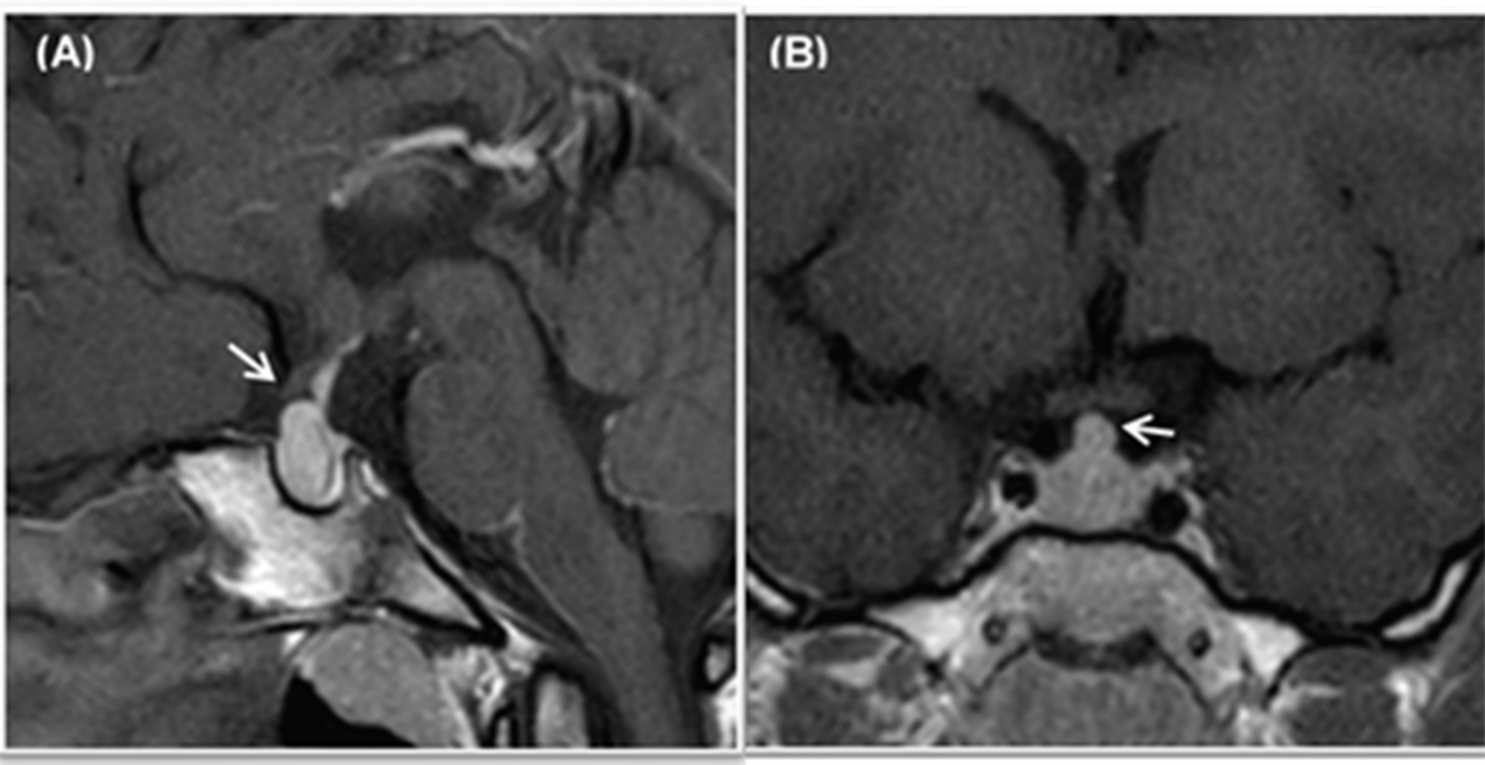
Pituitary gland Magnetic Resonance Imaging (MRI) after Gadolinium-DTPA administration of a patient at the time of diagnosis of pituitary hyperplasia and severe hypothyroidism. Sagittal (**a**) and Coronal T1-weighted images (**b**) that showed diffuse enlargement of the pituitary gland and a homogeneous enhancement of the gland. The enlarged pars tuberalis extended into the suprasellar cystern with mild compression of the optic chiasm (arrows)

Cerebral MRI documented symmetrical enlargement of the adenohypophysis in the entire cohort with a median pituitary volume of 770 mm^3^ (IQR 504–3528) (Table 2) and a homogeneous enhancement of the gland after the administration of Gadolinium-DPTA (Fig. 1). Moreover, no changes in bone structures near the pituitary gland were detected. No neurological symptoms (including headache and visual impairment) related to sellar expansion were reported in the entire cohort. No correlation was documented between TSH, FT4, FT3 levels and adenohypophysis volume, taking into account patients’ sex and age.

Once the diagnosis of hypothyroidism was made, gradual replacement therapy with levothyroxine was started, until a maintenance dose was reached (median maintenance levothyroxine dose of 2 mcg/kg/day, 0.6–3.5). Patients underwent close follow-up every 3 months. Ten patients continued the follow-up to 3-month (Table 3). Data at 6-month assessment were only available for 8 patients (Table 4). Nine patients continued the follow-up to 12-month (Table 5).

One patient (patient 2) experienced the onset of puberty three months after starting levothyroxine therapy. Moreover, symptoms and signs described at diagnosis have completely disappeared in 80% of patients at the 3-month assessment; patient 1 still had dry and thickened skin and patient 4 presented sparse brittle hair and dry and thickened skin too.

Laboratory tests showed normalization of thyroid function in 80% of patients (median and IQR: TSH 2.07 mUI/ml, 0.24–25.14; FT4 12.8 pmol/L, 9.39–17.24) at the 3-month assessment. IGF-1 values were lower than − 2 SDS in only one patient (patient 4) (median value 148 ng/ml; IQR: 138-195.3), instead PRL concentrations, measured in only 4 of the 10 patients, decreased (median value 88.1 uUI/ml) as shown in Table 3.

Two patients (patients 2 and 3) from the same Italian centre, in whom stunted height growth persisted after restoration of normal thyroid hormone levels, had GH peaks of less than 8 ng/ml in two tests performed with different stimuli to assess GH secretion. For these patients, it was decided to continue auxological follow-up for a further 6 months to assess spontaneous improvement in stature.

Moreover, three patients underwent brain MRI, which documented a partial regression of pituitary volume (patient 12) and a complete regression of pituitary hyperplasia (patient 6 and patient 7) as shown in Table 3.

**Table 3:** Clinical features, laboratory findings and pituitary volume at 3-month assessment

Patients	Height, cm (SDS)	Pubertal Stage (B/G-P)	Clinical findings	TSH, mUI/ml	FT4, pmol/l	TPOAb, IU/ml	TGAb, IU/ml	IGF-1, ng/ml	PRL, uUI/ml	Pituitary volume, mm3
Patient 1	133.7 (-3.72)	B3P1	Dry and thickened skin	0.24	12.3	NA	NA	NA	NA	NA
Patient 2	131.6 (-1.83)	B2P2	No symptoms	7.2	11.7	NA	NA	148.3	91.1	NA
Patient 3	124.2 (-1.08)	B1P1	No symptoms	2.47	17.24	NA	NA	NA	NA	NA
Patient 4	135.8 (-1.74)	G1P1	Sparse brittle hair, dry and thickened skin	25.14	9.39	633.2	502.44	138	406.38	NA
Patient 5	154.2 (-1.27)	G2P2	No symptoms	2.4	13.12	68.8	9.5	195.3	NA	NA
Patient 6	117.6 (-2.21)	B1P1	No symptoms	0.54	11.19	58.1	19.8	195	85.1	198.7
Patient 7	122 (-1.48)	B1P1	No symptoms	2.11	12.48	160.4	202.6	146.1	57.44	184.5
Patient 8	NA	NA	No symptoms	2.01	14.6	NA	NA	NA	NA	NA
Patient 11	160 (-1.06)	NA	No symptoms	0.2	15.4	NA	NA	NA	NA	NA
Patient 12	137.5 (-2.19)	NA	No symptoms	2.04	17.1	NA	NA	NA	NA	440.4

TSH = thyroid stimulating hormone; FT4 = free thyroxine; TPOAb = thyroid peroxidase antibodies; TGAb = thyroglobulin antibodies; IGF-1 = insulin growth factor-1; PRL = prolactin; M = male; F = female; NA = not available

At 6-month assessment, regular height growth velocity has been observed in all 8 patients who continued the follow-up with a median height of -1.59 SDS (Table 4). No new symptoms or clinical findings were reported, even if patient 1 still presented dry and thickened skin. Normal thyroid function has been observed in almost all patients (87%), even if laboratory tests showed values of TSH slightly above the upper limit of normal range in one patient (patient 12). The median value of TSH was 1.36 mUI/ml (IQR 0.2–6.42) with median value of FT4 of 15.62 pmol/l (IQR 12.9–19.3).

Four patients underwent brain MRI, with evidence of a marked reduction of pituitary volume in patient 4 and patient 11 and a complete regression of PH in patient 2 and patient 5 as shown in Table 4.

At 12-month assessment after the beginning of levothyroxine, an improvement in terms of height growth has been observed in 8 patients (88%) who continued the follow-up (Table 5). Their median height was − 1.08 SDS with a median increase of growth velocity of 10 cm/year. One patient (patient 4) experienced the onset of puberty, while patient 1 still had dry and thickened skin. Laboratory tests showed normalization of thyroid function in 8 patients who continued the follow-up and values of TSH slightly above the upper limit of normal range in one patient (patient 2). The median value of TSH was 1.6 mIU/L (IQR 0.2–5.62) and of FT4 was 15.44 pmol/L (IQR 11.32–17.25). A reduction of antibody title was also observed with a median value for TPOAb and TGAb of 205.35 IU/ml and 93.55 IU/ml respectively. The dose of levothyroxine was essentially stable over the year (2.15 mcg/kg/day; 1.8–3).

Levels of IGF-1 were in range for age and sex only in four patients (median value of 213.5 ng/ml), instead two patients (patient 4 and patient 5) presented levels lower than − 2 SDS. The two patients with a previous pathological response to stimulus tests to assess GH secretion had normalized height growth and hormone secretion. Moreover, evaluation of anterior pituitary hormone function, measured in only 3 of the 9 patients, showed normal PRL levels in all patients with median value of 127.63 u[IU]/ml.

Laboratory tests showed normal values of total and LDL cholesterol, with a median value of 143 mg/dl and 77.7 mg/dl, respectively. Normal levels of triglycerides (median value of 48 mg/dl), GOT and GPT (median value of 34.5 U/L and 12.5 U/L respectively) were documented.

The control brain MRI performed 1 year after diagnosis of PH documented complete regression of PH with a volume within the normal range for age and sex in five patients who continued follow-up as shown in Table 5.

**Table 4:** Clinical features, laboratory findings and pituitary volume at 6-month assessment

Patients	Height, cm (SDS)	Pubertal Stage (B/G-P)	Clinical findings	TSH, mUI/ml	FT4, pmol/l	TPOAb, IU/ml	TGAb, IU/ml	IGF-1, ng/ml	PRL, uUI/ml	Pituitary volume, mm3
Patient 1	136.7 (-3.5)	B4P2	Dry and thickened skin	0.2	17.37	NA	NA	NA	NA	NA
Patient 2	134.4 (-1.81)	B3P3	No symptoms	0.239	18.7	1755	291	182.2	89	287.0
Patient 4	139.1 (-1.5)	G1P1	No symptoms	0.4	14.9	371	323.2	146	NA	327.6
Patient 5	163.1 (-0.92)	G3P3	No symptoms	1.6	15.44	58.2	15.1	234.7	NA	171.6
Patient 6	122.1 (-1.69)	B1P1	No symptoms	1.12	19.3	48.1	21.8	195	110.63	NA
Patient 7	123.5 (-1.46)	B1P1	No symptoms	1.8	12.99	140.4	192.4	196.1	57.44	NA
Patient 11	163.5 (-0.73)	G5P5	No symptoms	1.66	15.8	NA	NA	NA	NA	563.16
Patient 12	140.8 (-2.01)	G4P3	No symptoms	6.42	12.9	NA	NA	NA	NA	NA

TSH = thyroid stimulating hormone; FT4 = free thyroxine; TPOAb = thyroid peroxidase antibodies; TGAb = thyroglobulin antibodies; IGF-1 = insulin growth factor-1; PRL = prolactin; M = male; F = female; NA = not available

**Table 5:** Clinical features, laboratory findings and pituitary volume at 12-month assessment

Patients	Height, cm (SDS)	Pubertal Stage (B/G-P)	Clinical findings	TSH, mUI/ml	FT4, pmol/l	TPOAb, IU/ml	TGAb, IU/ml		IGF-1, ng/ml		PRL, uUI/ml	Pituitary volume, mm3
Patient 1	139.8 (-3.25)	B4P4	Dry and thickened skin	0.2	17.25	NA	NA	NA		NA		NA
Patient 2	136.6 (-1.83)	B3P3	No symptoms	5.62	13.6	223	279	230		134		287.0
Patient 3	134.4 (-0.06)	B2P2	No symptoms	1.23	16.1	374	1114	445		157		160.3
Patient 4	145.1 (-1.1)	G2P2	No symptoms	3.1	11.32	191.7	72.1	125		NA		NA
Patient 5	169.1 (-0.39)	G4P4	No symptoms	1.4	15.05	48.5	19.2	120.3		NA		171.6
Patient 6	129.5 (-1)	B1P1	No symptoms	1.16	12.61	39.3	17.9	197		121.27		NA
Patient 7	129.1 (-1.06)	B1P1	No symptoms	2.38	13.77	219	115	183.2		57.44		NA
Patient 8	NA	NA	No symptoms	2.25	15.0	NA	NA	NA		NA		185.00
Patient 12	144.2 (-1.87)	G4P3	No symptoms	4.86	13.9	NA	NA	NA		173.6		270.4

TSH = thyroid stimulating hormone; FT4 = free thyroxine; TPOAb = thyroid peroxidase antibodies; TGAb = thyroglobulin antibodies; IGF-1 = insulin growth factor-1; PRL = prolactin; M = male; F = female; NA = not available

## Discussion

AT is the most common form of thyroiditis and common cause of acquired PHT in children and adolescents, although it is often characterized by a biochemical picture of euthyroidism or subclinical hypothyroidism [15–17]. Enlargement of the thyroid gland is the presenting sign in most children with AT. Growth delay is rare in autoimmune thyroiditis, if timely diagnosed. In our entire cohort, AT determined a severe hypothyroidism that clinically appears only with height growth arrest or growth impairment. In fact, no goiter has been documented due to the atrophic phenotype of AT. AAT is more common in adults than in children, where it occurs most frequently before puberty (peak of incidence from 6 to 9 years of age). AAT is typically characterized by lack of goiter development and, because of the delay in the diagnosis, by severe hypothyroidism and growth impairment [1–4]. AAT pathogenesis is very complex. An important role is played by TSH-receptor blocking antibodies (TSBAb) by blocking TSH-induced stimulation of thyroid growth and hormonogenesis. TSBAb positivity contribute significantly to the severity of hypothyroidism [18, 19]. TSBAb was not available in our retrospective casuistry.

A wide variability of PH incidence in patients with PHT has been reported (from 25 to 81%) [20]; adenohypophysis volume would seem to be able to be correlated with TSH levels [21]. A correlation between thyroid hormone levels and adenohypophysis volume has not been documented in our population. Approximately the 85% of PH’s cases regress after levothyroxine therapy administration [21]. In our case series, all patients who continued follow-up presented a normalization of thyroid hormone levels.

Thyroid hormone levels as a negative feedback regulatory signal affects the production of TSH. The lack of negative feedback in hypothyroidism causes excessive secretion of TRH, and conduce to a proliferation of TSH secreting cells, which results in compensatory enlargement of the pituitary gland (TRH-dependent hyperplasia). It has been suggested that TSH-releasing cells hyperplasia in PHT may be promoted by somatotroph to thyrotroph cell transdifferentiation [22]. Somatotroph and thyrotroph pituitary cells in mammals derive from the same precursor cell expressing the transcription factors Prop 1 and Pit 1 in ontogeny [23]. Bi-hormonal cells, expressing both GH and TSH, have been documented both in rat pituitary as in human pituitary adenomas. Furthermore, in normal pituitary cells as in adenoma, endogenous TRH and TRH receptors (TRHR) were documented not only in thyrotroph but also in somatotroph cells [24, 25]. According to these evidences, Radiana et al. [23], in a cohort of methimazole-induced hypothyroid rats, documented a significant increase in number of pituitary TSH-releasing cells. They also demonstrated in somatotroph cells the presence of TSH secretory granules, an increased TRHR-immunoreactivity and a reduced GH immunoreactivity. In light of these findings, these authors suggested that the increased number of TSH-releasing cells was due, at least partially, to the transdifferentiation of somatotroph into thyrotroph cells, likely promoted by TRH stimulation. These somatotroph-derived thyrotrophs cells, characterized by a bi-hormonal pattern, have also been detected in adenohypophysial biopsies from humans with protracted PHT in whom the role of transdifferentiation in PH has been confirmed [26]. Transdifferentiation is a reversible process, therefore the resolution of PHT lead to disappearance of bi-hormonal cells and reappearance of somatotrophs [22]. Bi-hormonal transitional cells are expression both of the multipotential pattern of pituitary cells, with respect to hormonogenesis, and of the need of specific hormone increased secretion. Therefore, we suggest that these evidences might explain the transient GH deficiency revealed in our two patients, that spontaneously solved after the beginning of levothyroxine replacement therapy.

Thyroid hormones influence not only TSH gene expression, with a negative feedback mechanism, but also GH gene expression. Particularly, a dose-related effects of triiodothyronine administration in stimulating GH gene expression and inhibiting TSH gene expression have been demonstrated in thyroidectomized rats [27]. These opposite effects of T_3,_ both on GH and TSH gene expression, are explained by the T_3_-induced interaction between nuclear T_3_ receptors (TR) and TR-responsive elements in the 5’-flanking regions of both GH and TSH genes [28]. Furthermore, Childs et al., in propylthiouracil-induced hypothyroid rats, demonstrated an increased expression of TR-β2 mRNA in TSH-releasing cells and its decreased expression in GH-releasing cells, these latter significantly reduced in number in absence of circulating T_3_ [29].

Moreover, TRH also has a weak stimulatory effect on lactotroph cells, responsible of the state of hyperprolactinemia (lactotroph hyperplasia) as also observed in our patients [20]. High levels of PRL could be also secondary to reduced hypothalamic dopamine content due to compression of hyperplastic pituitary gland on the pituitary stalk [30]. These findings further clarify the complete and rapid restoration of normal GH secretion and PRL concentrations in our cohort when euthyroidism was restored.

Moreover, in one of our patients (patient 12) a clinical diagnosis of Van Wyk and Grumbach syndrome was made [13, 14]. This condition is identified by a phenotype characterized by delayed bone age and pseudoprecocious puberty in the context of severe hypothyroidism and pituitary hyperplasia. The pathogenesis of this condition is closely associated with the interaction between gonadotropins and TSH, which share a common α-subunit [13, 14]. High levels of TSH may act as an FSH receptor agonist resulting in increased testicular volume. In addition, the increase in testicular volume may be promoted by increased FSH levels (associated with decreased LH) caused by a decrease in GnRH pulse rate and downregulation of its secretion, which sometimes occurs in severe hypothyroidism [13, 14].

PH due to PHT should be considered in the differential diagnosis of height growth arrest. PH may cause a temporary deficit of the pituitary hormones secretion and, in particular, a GH secretion impairment due, at least partially, to TRH-induced transdifferentiation of somatotroph into thyrotroph cells. In patients with pituitary enlargement, thyroid function tests are important to recognize PH secondary to PHT and to avoid unnecessary surgery.

The retrospective design, as well as the small sample size, represent limitations of our study. Furthermore, the lack of data on pelvic and testicular ultrasounds represents another limitation of the study. In addition, TSBAb assay and values of some hormones in the follow-up were not available due to retrospective evaluation of the case series.

## Conclusions

This is the largest pediatric cohort with severe autoimmune PHT without goiter but with pituitary hyperplasia in which significant growth impairment was the most evident presenting sign. Atrophic AT phenotype might be correlated with this specific clinical presentation. Since thyroid hormones promote GH biosynthesis, GH deficiency could be expected proportional to the hypothyroidism degree in severe PHT. However, in some hypothyroid patients IGF-1 concentration may be within normal range even though they have growth impairment. In youths with growth impairment, hypothyroidism should always be excluded even in the absence of clear clinical signs of dysthyroidism.

### Electronic supplementary material

Below is the link to the electronic supplementary material.

Supplementary Material 1

## Data Availability

The datasets used and/or analysed during the current study are available from the corresponding author on reasonable request.

## Abbreviations

AT: Autoimmune thyroiditis
PHT: Primary hypothyroidism
AAT: Atrophic autoimmune thyroiditis
PH: Pituitary Hyperplasia
CT or MRI: brain computed tomography or magnetic resonance imaging
TPOAb: free triiodothyronine or FT3 and free-thyroxine or FT4, thyroid peroxidase antibodies
TGAb: thyroglobulin antibodies
ACTH: adrenocorticotropic hormone
PRL: prolactin
FSH and LH: follicle stimulating hormone and luteinizing hormone
IGF-1: like growth factor-1
GOT: oxaloacetate transaminase
GPT: pyruvate transaminase
