# Supplementary material for: Growth impairment in children with atrophic autoimmune thyroiditis and pituitary hyperplasia
Source: Ital J Pediatr. 2024 Apr 23;50:83. doi: 10.1186/s13052-024-01641-w (PMC11036550; doi:10.1186/s13052-024-01641-w)
Supplement: Supplementary file 1 — Supplementary Material 1 [file 13052_2024_1641_MOESM1_ESM.pdf]

Table. S1 Normal values of IGF-1 (ng/ml) for age and for sex

Age (years)	Male	Female
0-1	12-94	15-89
1-3	15-105	26-129
4-6	37-158	53-217
7-9	68-255	87-324
10-11	90-368	118-413
12-14	108-463	139-466
15-17	15-500	154-483
18-20	134-449	154-448
